# Seroprevalence of parvovirus B19 IgG in children affected by juvenile idiopathic arthritis

**DOI:** 10.1186/ar2281

**Published:** 2007-08-30

**Authors:** Benedikt Weissbrich, Yvonne Süß-Fröhlich, Hermann J Girschick

**Affiliations:** 1 Institute of Virology and Immunobiology, University of Würzburg, Versbacher Str 7, 97078 Würzburg, Germany; 2 Section of Paediatric Rheumatology, Immunology and Infectious diseases, Children's Hospital, University of Würzburg, Josef-Schneider-Str 2, 97080 Würzburg, Germany

## Abstract

Parvovirus (PV) B19 is the causative agent of the childhood disease erythema infectiosum. An association of PV B19 with chronic arthropathies, sometimes resembling rheumatoid arthritis or juvenile idiopathic arthritis (JIA), has repeatedly been described. Other studies, however, have failed to identify any such relationship. In order to study further whether there is a link between PV B19 and JIA, we determined the prevalence of PV B19 specific IgG antibodies in serum samples from children with rheumatoid diseases and compared it with the prevalence in unaffected children We reasoned that if there is an association between PV B19 and JIA, then the prevalence of PV B19 IgG in the children with JIA should be higher than in the control group. PV B19 IgG status was tested in 406 children with JIA and related diseases, and in 146 children constituting a control group. The percentage of PV B19 IgG positive children was not significantly elevated in the disease subgroups compared with age-matched control groups. In conclusion, our findings do not support the hypothesis that human parvovirus B19 is involved in the pathogenesis of JIA.

## Background

Parvovirus (PV) B19, the causative agent of the childhood disease erythema infectiosum (fifth disease), was identified in 1975. Since then a large spectrum of diseases caused by or associated with PV B19 has been recognized (for review [1,2]). In addition to erythema infectiosum, nonspecific febrile illnesses and asymptomatic courses are common. Furthermore, the clinical spectrum of PV B19 includes haematological, neurological and cardiovascular manifestations. Infection during pregnancy may result in hydrops fetalis. Arthralgias and acute arthritis are well known complications of acute PV B19 infection in children and in adults [3].

An association of PV B19 with chronic arthropathies, sometimes resembling rheumatoid arthritis or juvenile idiopathic arthritis (JIA), has also been described, but other studies have been unable to corroborate these findings. It therefore remains unclear whether there is an aetiological or pathogenic link between PV B19 and rheumatoid arthritis or JIA (for review [4]).

The term JIA encapsulates a heterogeneous group of rheumatic diseases, which – for most subtypes – is distinct from adult rheumatoid arthritis [5]. Manifesting as early as in the first year of life, childhood arthritis can be a serious disease that affects not only motor neurone but also psychosocial development. Autoimmune features have been noted especially in two subgroups, namely enthesitis related arthritis and early onset pauciarticular arthritis (EOPA). Infectious diseases have long been suspected as being trigger factors for the initial manifestation of arthritis and subsequent flare ups, and various bacteria and viruses have been implicated in this regard [6-10]. In particular, Still's disease in childhood has been associated with PV B19; in fact, in some children affected by Still's disease the erythematous rash resembles that of erythema infectiosum [11,12].

In order to evaluate further the possible link between PV B19 and JIA, we determined the prevalence of PV B19 specific IgG antibodies in serum samples from children with rheumatic diseases and compared it with the prevalence in unaffected children. Whereas PV B19 IgM is detectable only for about 1 to 3 months after an acute infection, PV B19 IgG persists throughout life. Thus, the presence of PV B19 IgG is a marker of previous exposure to PV B19. We hypothesized that if there is an association between PV B19 and JIA, then the prevalence of PV B19 IgG in children with JIA should be higher than in the control group.

## Materials and methods

### Patients

The study population consisted of children who were referred to the Section of Paediatric Rheumatology at the University of Würzburg, Germany, between 1988 and 2001. Serum samples for routine laboratory studies were obtained from the patients at the initial visit. No selection of patients was performed. Unused serum was kept frozen at -20°C. Demographic data, clinical diagnosis, and information on PV B19 status were extracted from the records. Stored serum samples of children with unknown PV B19 status were retrospectively tested for PV B19 IgG.

In addition, serum samples of 146 children were analyzed as controls. These samples were collected from children referred to the Endocrinological Section of the Children's Hospital and from children referred to the Section of Paediatric Rheumatology who presented with complaints not associated with arthropathy or infections.

All patients and control children were of white Caucasian descent. The study was conducted in compliance with the Helsinki Declaration and was approved by the ethics committee at the University of Würzburg. Informed consent was obtained from the parents or legal guardians of the children in the arthritis group for standard of care diagnostic procedures, which included serology on infectious agents associated with arthritis. For the control group, the use of anonymized residual serum samples obtained for routine diagnostic procedures was approved by the ethics committee.

### Parvovirus B19 serology

PV B19 IgG antibodies were determined using an indirect immunofluorescence assay. Briefly, SF9 insect cells infected with baculovirus recombinant for VP1 of PV B19 were spotted on glass slides, air dried, fixed with cold acetone and stored at -20°C until use. For IgG determination, the fixed cells were incubated for 90 min with a fourfold dilution series of each plasma sample, starting at 1:10. Subsequently, the slides were washed with phosphate-buffered saline and incubated with fluoresceine-conjugated goat-anti-human-IgG (Medac, Hamburg, Germany) and Evans blue for 30 min. After another washing step, coverslips were mounted for immunofluorescence microscopy. The IgG slides were independently read by two experienced persons. If the results did not match, a third person read the slides and the median value was used for further analysis. Samples with a PV B19 IgG titre above 1:10 were counted as positive.

### Statistical analysis

Fisher's exact test was used to compare the seroprevalence proportions of PV B19 between different groups, and the Mann-Whitney test was used to compare PV B19 IgG titers. *P *≤ 0.05 was considered statistically significant.

## Results

### Patients and control children

**Figure 1:**
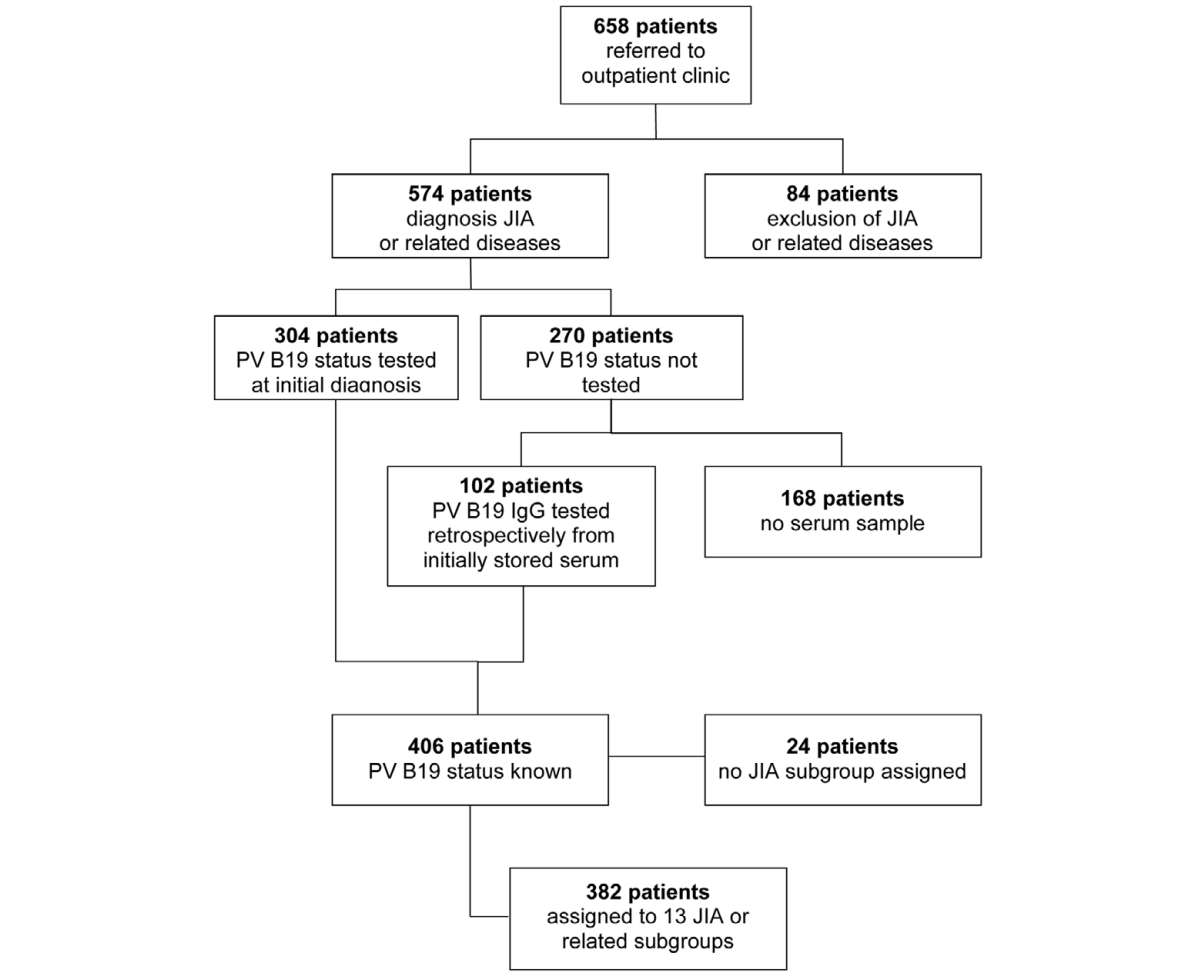
Enrollment, JIA diagnosis and PV B19 testing. JIA, juvenile idiopathic arthritis; PV, parvovirus.

Between 1988 and 2001, 658 children were referred to the section for paediatric rheumatology. JIA or related rheumatic diseases were diagnosed in 574 children. In 84 of the children, no rheumatic disease was diagnosed. The PV B19 IgG status had already been determined as part of the routine check up in 304 of the children. Of the remaining 270 children in whom information on PV B19 serostatus was not available, stored serum samples were available for 102. Both the children with documented PV B19 status from the initial diagnosis and children in whom PV B19 status was determined retrospectively were included in the analysis (Figure 1). Children were classified into subgroups of JIA or related rheumatic diseases based on the Durban Criteria of the International League of Associations for Rheumatology [13]. A minor modification was used. Because EOPA is considered to be a separate disease entity, we differentiated this subgroup from other oligoarthritides. The subgroups of children, along with demographic data, are summarized in Table 1. Twenty-four children were excluded from the study because retrospective classification ruled out rheumatic or associated diseases. In order to allow age-matched comparison of PV B19 IgG status, the control group was randomly divided into three subgroups with mean ages and age ranges comparable to those in the patient subgroups (Table 1).

**Table 1:** Demographic data and PV B19 serostatus of the disease subgroups and the corresponding age-related control subgroups

Classification	Disease subgroups^a^						Age-matched control groups					
	
	*n*	Female (%)	Mean age (years)^b^	Age range (years)^b^	B19 IgG^+ ^(*n *[%])	*P*^c^	Subgroup	*n*	Female (%)	Mean age (years)^b^	Age range (years)^b^	B19 IgG^+ ^(*n *[%])
EOPA	67	76.1	4.5	1.3–15.0	21 (31.3)	<0.05	1	36	38.9	5.5	0.8–9.3	17 (47.2)
Systemic arthritis (Still's disease)	8	100	6.7	4.8–11.1	5 (62.5)	NS						
Eye disease, rheumatoid (iridocyclitis)	6	83.3	7.3	3.8–11.1	2 (33.3)	NS						
Reactive arthritis	38	31.6	7.5	1.8–16.2	15 (39.5)	<0.05	2	30	50.0	8.9	1.5–15.8	19 (63.3)
Other arthritis (unclassified)	28	50	8.8	1.0–15.1	16 (57.1)	NS						
Arthralgias	85	48.2	9.3	2.3–16.5	53 (62.4)	NS						
Polyarthritis (RF^+ ^and RF^-^)	19	78.9	9.7	2.6–15.7	11 (57.9)	NS						
Lyme arthritis	37	43.2	10.2	2.8–15.5	25 (67.6)	NS	3	80	41.3	11.2	1.9–18.3	52 (65.0)
Other oligoarthritis (non-EOPA JIA)	13	38.5	10.6	4.8–18.8	7 (53.9)	NS						
Psoriatic arthritis	11	63.6	10.7	1.3–15.0	7 (63.6)	NS						
CRMO	12	66.7	11.5	6.8–15.3	6 (50.0)	NS						
EAA	54	61.1	11.6	1.9–18.3	39 (72.2)	NS						
SLE	4	100	13.0	9.7–15.9	4 (100)	NS						

^a^Subgroups ordered by mean age. ^b^Age when serum sample used for parvovirus (PV) B19 testing was drawn. ^c^Comparison of disease subgroup with age-matched control group. CRMO, Chronic recurrent multifocal osteomyelitis; EAA, Enthesitis associated arthritis; EOPA, Early-onset pauciarticular arthritis; JIA, juvenile idiopathic arthritis; NS, not significant; RF, rheumatoid factor; SLE, System ic lupus erythematosus.

### Results of PV B19 serology

The results of PV B19 IgG determination are shown in Table 1. The percentage of PV B19 IgG positive children was not significantly elevated in the disease subgroups compared with the age-matched control groups. In fact, for the EOPA-JIA subgroup, the percentage of PV B19 IgG positive children was significantly lower than in the control group.

Individual virus specific IgG titres are influenced by various factors, which prevents definition of normal values. Therefore, comparison of IgG titres between different groups is usually of only very limited value. For completeness, IgG titres of the PV B19 IgG positive children were compared between the major JIA subgroups (enthesitis associated arthritis, EOPA-JIA and polyarthritis). No significant differences were identified (data not shown).

## Discussion

Comparing the seroprevalence of PV B19 IgG between children with JIA and a control group, we found no significant difference in PV B19 seropositivity. Our study was based on the assumption that if there is an association between PV B19 and a substantial proportion of JIA cases, then the prevalence of PV B19 IgG in the children with JIA should be higher than that in the control group, irrespective of time of infection. However, our findings do not support an association between PV B19 and JIA.

In general, there are two basic approaches to diagnosing viral infections: application of serological tests for antibody detection and application of direct viral detection methods (in particular polymerase chain reaction). Both approaches have been used to study the potential link between B19 and JIA, but thus far no unequivocal evidence favouring or refuting such an association has been presented.

Following a description of a case of juvenile chronic arthritis following acute infection with PV B19 [14], an observational study conducted in 22 children with joint complaints in conjunction with a recent PV B19 infection [12] identified six children who developed chronic arthritis for 2 to 13 months. Although the arthritis was attributed to the PV B19 infection, these children would also have fulfilled the diagnostic criteria for juvenile rheumatoid arthritis (JRA) [15].

In a study conducted in Japan [16], adult as well as juvenile patients with rheumatoid arthritis were tested for B19 IgG. Out of four patients with Still's disease, none was positive for PV B19 IgG. However, five out of 11 patients (mean age 19.1 years, range 12 to 27 years) with polyarticular JRA (according to the criteria of the American Rheumatism Association of 1976 [15]) were positive for B19 IgG, as compared with five out of 60 age-matched control patients. The difference was statistically significant (*P *= 0.006, by Fisher's exact test), but the authors expressed concern in their discussion that statistical bias from case sampling might have occurred. Furthermore, the number of patients with JRA included in the study was low. In addition, the mean age was high, suggesting blood sampling later in the course of disease.

In a study conducted in India [17], 69 children and adolescent persons with JRA (according to the criteria of the American Rheumatism Association [18]), 26 adults with rheumatoid arthritis (disease control individuals) and 12 healthy children were tested for PV B19 IgG and IgM. Although the proportion of PV B19 between patients (35/69 [50.7%]) and control individuals (19/38 [50.0%]) was almost identical, PV B19 IgM was found significantly more often in the JRA group. Although detailed information on the distribution of possible constellations of PV B19 IgG and IgM antibodies was not provided in the results section of the report, it can be deduced from the abstract that ten JRA patients (14.5%) were positive for PV B19 IgM but negative for PV B19 IgG. Because none of the patients had clinical symptoms suggestive of acute PV B19 infection, this finding is highly unlikely. Thus, the specificity of the PV B19 IgM test used in this study must be questioned.

Oguz and coworkers [19] used a different serological approach to study the potential association between PV B19 infections and JRA. They determined PV B19 IgM in 75 children with acute arthropathy. Sixteen were found to be positive for PV B19 IgM. Children with persisting joint complaints were followed up for at least 6 months. Three of the PV B19 IgM positive patients but only one of the PV B19 IgM negative children were diagnosed with JRA (*P *= 0.03). Prospective studies such as this one are important in determining whether there is a potential risk for developing JRA after acute PV B19 infection. Unfortunately, acute PV B19 infections in this study were diagnosed solely using a peptide-based PV B19 IgM enzyme immunoassay. Similar peptide-based assays have been found to yield a considerable number of false-positive results compared with assays based on recombinant antigens expressed in the baculovirus system, especially in the presence of autoantibodies and rheumatoid factor [20-22]. Confirmation of the findings presented by Oguz and coworkers would therefore be desirable.

In an Italian study, Angelini and colleagues [23] compared the prevalence of PV B19 IgG in a group of 35 children fulfilling the 1987 criteria of the American College of Rheumatology for rheumatoid arthritis [24] with that in a control group of 93 children. The difference between the two groups was significant (PV B19 IgG positive: 45.7% in the arthritis group versus 24.7% in the control group). The number of cases was considerably smaller than that in the present study. Furthermore, the patient and control populations cannot be compared with those included in our study. Whereas we applied the Durban criteria of juvenile idiopathic arthritis of 1997 to select our patients [13], Angelini and colleagues used the criteria of the American College of Rheumatology to diagnose JRA. The control population in their paper is not described in sufficient detail to allow a comparison.

In another study of PV B19 and juvenile arthritis, Lehmann and coworkers [25] examined PV B19 DNA in serum of 48 children with joint complaints, which were selected on the basis of positive IgG antibodies against the PV B19 nonstructural protein (NS)1. Fifteen patients were positive for PV B19 DNA, as compared with nine out of 124 healthy control children. However, only 27 of the control children were positive for NS1 IgG, and three of these were positive for PV B19 DNA. Assuming that the NS1 IgG positive children constitute the appropriate control group, the proportions of PV B19 DNA positive children were not significantly different between patients and control children. In a follow-up study with the same control group but including consecutively enrolled hospitalized children with rheumatic diseases [11], the proportions of PV B19 IgG positive children were compared between patients and control children. In agreement with our findings, there was no significant difference. However, both NS1 IgG and PV B19 DNA were found more frequently in serum samples of children with rheumatoid disease. PV B19 DNA was also detected in 22% of synovial membrane samples of children with JIA. However, a control group for comparison was not assessed. In consideration of the fact that the overall frequency of PV B19 infections between patients and control individuals exhibited no difference, it remains to be determined how the greater frequency of PV B19 DNA in serum of children with JIA could reflect pathogenicity of PV B19 for rheumatoid diseases. Further studies will be necessary to prove that the presence of PV B19 DNA in serum of children with rheumatoid disease is not merely an epiphenomenon.

This issue is also pertinent to several other studies, both in adults and in children, that described detection of PV B19 DNA in synovial fluid, cells, and tissue [26-32]. However, because PV B19 DNA is also found in control samples with varying frequency, it is still unclear whether the presence of PV B19 DNA in synovial material is of pathogenic relevance [26,27,29,30]. It was recently suggested that PV B19 DNA may persist in human tissues throughout life [33]. Thus, the presence of PV B19 in synovium appears insufficient in terms of proving that it causes arthritis [34].

Because of its retrospective nature, our study has limitations with respect to case finding and description of clinical manifestations. Not all case patients were tested for PV B19 IgG. However, there is no indication of selection bias caused by the exclusion of children for whom stored serum samples were not available. PV B19 polymerase chain reaction and PV B19 IgM tests were not used in this study because of limitations inherent in the interpretation of positive results obtained with these methods, and because the hypothesis to be tested in this study was based on PV B19 IgG status.

## Conclusion

In summary, there is no conclusive evidence yet for a pathogenic role of PV B19 in JIA. Analysis of the seroprevalence of anti-PV B19 IgG antibodies in European Caucasian children affected by arthritis did not support the hypothesis that human PV B19 is involved in the pathogenesis of JIA.

## Abbreviations

EOPA, early onset pauciarticular arthritis; JIA, juvenile idiopathic arthritis; JRA, juvenile rheumatoid arthritis; NS, nonstructural protein; PV, parvovirus.

## Competing interests

The authors declare that they have no competing interests.

## Authors' contributions

YSF collected the patient data from the clinical records, performed the PV B19 IgG testing, and analyzed the data. BW and HJG designed and coordinated the study. BW contributed to the PV B19 testing and data analysis, and drafted the manuscript. HJG cared for the patients, participated in the analysis of the clinical data and contributed to the manuscript. All authors read and approved the final version of the manuscript.
